# Technology Access, Digital Literacy, and Enrollment Support Preferences in a Federally Qualified Health Center: Cross-Sectional Study

**DOI:** 10.2196/78850

**Published:** 2026-01-05

**Authors:** Katrina Go Yamazaki, Lucy Hewitt, Luis Torres, Kharla Colon-Vazquez, Peyton Rogers, Grace Wang

**Affiliations:** 1 Weitzman Institute, Moses-Weitzman Health System, 19 Grand Street, Middletown, CT, 06457, United States, 1 475 301 4516

**Keywords:** digital literacy, technology access, participant engagement, high-touch support, federally qualified health center

## Abstract

**Background:**

Biomedical research studies are increasingly using digital tools to enroll, recruit, and collect data from participants. However, variability in digital literacy and technological acceptance can be challenging for recruitment from groups traditionally underrepresented in research, including those served by Federally Qualified Health Centers.

**Objective:**

This study aimed to (1) measure participant accessibility and comfort with digital platforms and (2) examine the interrelation of technology access, digital literacy, and support preferences during enrollment and data submission.

**Methods:**

A cross-sectional analysis was conducted using enrollment data from Federally Qualified Health Centers participating in the All of Us Research Program. Participants had the option of High-Touch (staff-assisted) or Low-Touch (self-directed) support for enrollment and survey completion. Survey items assessed internet access and technology comfort, while support type was recorded by the research staff based on participants’ actual selection. Logistic regression models evaluated relationships between technology access, comfort, and enacted support type, while controlling for age, consent language, and education, as well as race and ethnicity.

**Results:**

The analytic sample included 605 participants. The majority reported access to the internet (539/605, 89.1%) and felt comfortable with technology (448/605, 74.1%). In the group requesting High-Touch support (n=346), 14.5% (n=50) reported no internet access, and 31.5% (n=109) felt uncomfortable with technology. In the group requesting Low-Touch support (n=259), 6.2% (n=16) had no access to the internet, and 3.9% (n=10) reported feeling uncomfortable (*P*<.001). In the adjusted models, much greater comfort with technology was significantly correlated with reduced odds of requesting High-Touch support (comfortable: adjusted odds ratio 0.118, 95% CI 0.055‐0.255 and neutral: adjusted odds ratio 0.212, 95% CI 0.077‐0.587), but internet access was not significantly correlated.

**Conclusions:**

The strongest predictor for support preference for digital enrollment among the participants was their comfort with technology rather than access alone. These findings illustrate the significance of participant-centric design methods coupling adaptive support paths, mixed enrollment strategies, and individualized onboarding methods aligned with digital confidence to promote equitable engagement in precision health research.

## Introduction

Biomedical studies should engage diverse populations to ensure findings are generalizable and advances in medicine are inclusive and accessible [1,2]. Historically, research participation has been disproportionately high among certain demographics, thereby creating inequities in prediction, prevention, and treatment [3,4]. Genome-wide association studies, for example, have focused predominantly on European-derived populations, representing around 78% of worldwide datasets, thus limiting their generalizability to other populations [4]. These disparities highlight the need for inclusion on multiple levels, recognizing that both biological determinants as well as social determinants of health impact equal participation in research [3-9].

Disparities also arise from the digital tools and methodologies used for participant engagement and recruitment in research. An increasing number of large-scale research studies are transitioning toward the use of digital technologies, including electronic health records (EHRs), apps, wearables, and web platforms, to facilitate recruitment, remote engagement, and remote collection of data [10,11]. Although these tools can reduce geographic barriers, optimize study logistics, and enable decentralized clinical trials, their effectiveness is contingent upon participants’ access to devices, internet connectivity, and comfort with technology [10,12-14]. However, numerous studies indicate variability in both the willingness and ability of individuals to use digital platforms, especially for activities such as digital consent, which raises concerns that technological advancements alone could exacerbate existing disparities [10-12].

The digital divide, defined by gaps in device ownership, internet access, and digital literacy, is one of the biggest barriers for equitable engagement in this digital arena [11]. The digital divide disproportionately impacts disadvantaged older adults, those from lower socioeconomic backgrounds, and those with limited digital literacy [11,15]. For initiatives such as the National Institutes of Health’s (NIH) All of Us Research Program (AoURP), which relies heavily on web-based consent, surveys, and remote biospecimen collection, these disparities risk reinforcing exclusion rather than fostering equity [11,15]. Studies across diverse communities, including Latino [16], Asian American [16], and other underserved communities [17,18], as well as among older adults [19], demonstrate persistent gaps in digital readiness. While such studies report barriers, few measure the extent to which digital access, literacy, and comfort correlate with participants’ support preferences during enrollment. For example, a High-Touch support approach is one where a staff member facilitates the consent and survey process, as well as provides the digital tools needed to complete the enrollment process. A Low-Touch approach is when the participant has access to the digital tools and is able to complete the enrollment process completely independently. Likewise, variation among Federally Qualified Health Centers’ (FQHCs’) patient portal enrollment processes accentuates the lack of unified methods to allow for digital readiness [20]. This represents an important gap. Without data on the relationship between readiness and support needs, programs risk misaligning strategies from the desired expectations among their participants, with the potential result that participation is inequitable.

To frame these relationships, this study is guided by the Digital Divide Framework, which details digital participation inequalities under 3 interconnected dimensions: structural (device and internet access), cognitive (ability and literacies to make use of digital tools), and motivational (attitudes, trust, and readiness to make use of technology) [21,22]. Under this framework, comfort with technology is conceptualized as an indication of digital self-efficacy, reflecting both skill and confidence in using digital tools. This aligns with constructs from the technology acceptance model (TAM) and the unified theory of acceptance and use of technology (UTAUT), both of which link perceived ease of use and confidence to adoption of digital systems [23,24]. By locating technological comfort within the theoretical constructs, this study examines how digital literacy and psychosocial factors interactively impact the study participants’ tendencies toward High-Touch from Low-Touch enrollment modes.

It is necessary to design participant-focused digital systems that are able to support varying digital readiness. Flexible support models, from the self-managed (Low-Touch) level to staff-assisted models (High-Touch), have the potential to ensure equal participation across varied populations [15,21,22]. This study used data collected during enrollment in the NIH’s AoURP, a national initiative designed to advance precision medicine by building one of the largest databases of participant-provided information, biospecimen samples, and DNA sequencing data. For this analysis, we focused on participants enrolled at Community Health Center Inc (CHCI), the largest FQHC in Connecticut.

Grounded in the Digital Divide Framework, we analyze the influence of structural, cognitive, and motivational factors on the support need, so as to develop strategies for fostering inclusive digital participation among FQHCs and other underrepresented groups, and supporting the equity objective of national-scale precision medicine initiatives such as AoURP. We hypothesized that participants who reported lower comfort with technology, which reflects lower digital literacy and self-efficacy, would be significantly more likely to prefer a High-Touch, or staff-assisted, enrollment support, even after accounting for internet access and sociodemographic characteristics.

## Methods

### Study Design

This cross-sectional study analyzed secondary data collected during enrollment into the NIH’s AoURP. The study followed the STROBE (Strengthening the Reporting of Observational Studies in Epidemiology) guidelines for reporting observational research [25].

### Study Setting

The NIH’s AoURP is dedicated to optimizing precision medicine by instituting the most complete database, comprised of information provided by the patient, biospecimen banks, and DNA sequencing data. Nationally, enrollment activities and retention were conducted over the full spectrum of sites, including the community-based activities, the primary care clinics, the academic medical centers, and FQHCs. The study targeted the enrollments from CHCI, the largest FQHC in the state of Connecticut. CHCI’s patient base is predominantly made up of low-income individuals (with 89% of the patient base living below the 200% federal poverty level) and is racially and ethnically mixed (61% Hispanic or Latino, 13.5% Black and African American, and 2.8% Asian).

### Participants

Eligible participants were more than 18 years old, able to provide independent consent, and resided in the United States. Enrollment into CHCI’s AoURP occurred between 2017 and 2023. CHCI offers services to the predominantly urban Connecticut area, where the participants are likely to have closer access to the broadband network compared to rural areas, which is relevant given known associations between rurality, internet access, and digital literacy. The participants whose data were incomplete were not considered, giving rise to potential selection bias if the participants whose data were incomplete systematically varied on digital access or digital comfort. The participants were not sampled by health condition nor by any previous exposure to technologies.

### Recruitment and Enrollment

Outreach was done by telephone calls as well as face-to-face contact, such as in the waiting rooms of the clinics. The enrollment process had participants enroll on the AoURP online platform, where they viewed and signed online informed consent documents, including general, EHR, as well as optional genomic consent. Participants completed 6 questionnaires on environmental factors, lifestyle, medical history, health care use, and digital readiness. Physical measures as well as biospecimens were also collected. The survey data and biospecimen data were linked by a single ID.

### Enrollment Support Models

**Figure 1.**
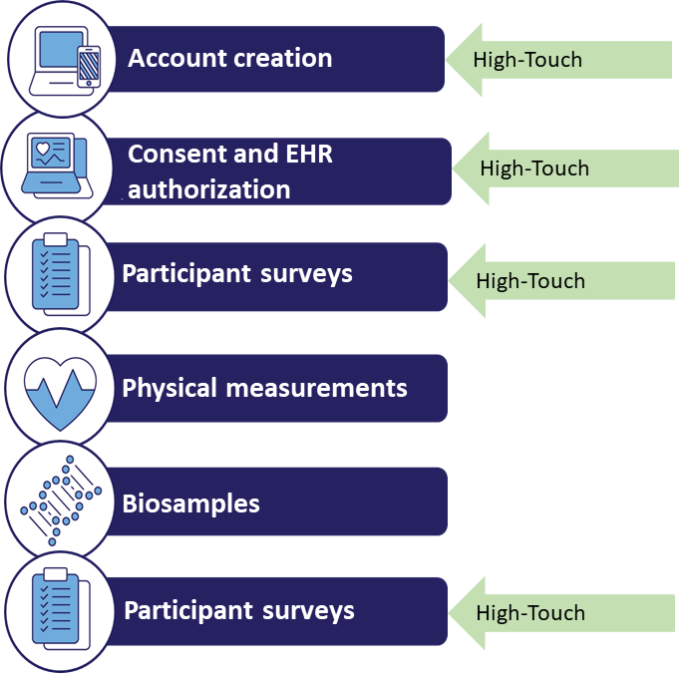
Patient journey. Enrollment process for the All of Us Research Program and places where the High-Touch strategy is implemented. EHR: electronic health record.

Participants were able to choose different levels of support during the enrollment process. Under the High-Touch model (Figure 1), AoURP staff helped participants enter their answers into the corresponding systems. The staff also helped participants gain access to necessary technologies, including tablets and internet connectivity, while also providing remote support by computer-assisted telephonic interviews, videoconferencing, and screen sharing. On the other hand, under the Low-Touch method, the participants filled out the forms and questionnaires by themselves, either on-site through the use of a tablet or off-site on their own device after receiving the call from the staff. The AoURP staff recorded the particular level of support used by the participants.

### Variables

The main outcome was the preference for High-Touch over Low-Touch support by the participant. Independent variables were internet access (yes and no) and level of tech comfort (comfortable, neutral, and uncomfortable). Covariates included age, gender, race and ethnicity, level of education, and consent language.

### Measures

Comfort with internet access and technology was assessed by survey questions completed during enrollment by the AoURP. They were prompted by the question “Do you have access to the internet at home?” (multiple response answers: yes, no, sometimes [<50% of the time], sometimes [>50% of the time], unknown or not reported, prefer not to answer). This question was very specific for defining internet access on any device at home and did not account for access at work, school, or other public locations. Internet access was dichotomized during analysis as “yes” versus “no” only, excluding “sometimes,” “unknown,” and “prefer not to answer” answers.

Comfort with technology was also measured with the question: “How comfortable are you using technology?” (very comfortable, somewhat comfortable, neutral, somewhat uncomfortable, not at all comfortable, prefer not to answer). Comfort with technology was combined into 3 groups: “comfortable” (very or somewhat comfortable), “neutral,” and “uncomfortable” (somewhat or not at all comfortable). The individual item on technology comfort, although self-reported, is used as a pragmatic proxy measure for digital literacy and self-efficacy. There is potential for future studies to consider proven multi-item scales from TAM and UTAUT to assess perceived ease of use and behavioral intention to use digital systems.

These scales were constructed for practice purposes between varied enrollment sites and are not standardized psychometric scales. While objective measures of technical proficiency (ie, timed exercises and direct observation of device usage) were not available, the self-report items provide pragmatic, standardized measures of participants’ reported digital literacy and self-efficacy. The adoption of self-report entails potential recall bias and social desirability bias, identified as a study limitation. Future research can incorporate objective digital proficiency measures or device metrics as an augment to reported comfort.

### Sample Size and Power Considerations

There was no a priori sample size calculation conducted as the data were observational. Post hoc calculation found >80% power to detect odds ratios (ORs) ≥2 among dichotomous predictors at *α*=.05. Smaller effects, particularly among small case subgroups (eg, no internet access), may not have been detectable.

### Analysis

Descriptive statistics (frequencies and percentages) were made for participant characteristics, access to technological tools, and technological comfort. Bivariate associations between internet access, technologic comfort, and type of support preferred (High-Touch vs Low-Touch) were examined by chi-square test. The key predictors were internet access, technological comfort, age, race and ethnicity, education, and language given for consent. The interactions were included to test the influence of technological comfort changing by age, race and ethnicity, education, or consent language.

Due to the scarce data present in particular categories, a penalized maximum likelihood estimate including Firth correction for bias was conducted by logistic regression (LR) using PROC LOGISTIC. The model included the key effects of internet access, technology comfort, age, race and ethnicity, education level, and the consent language, alongside 2-way interactions between technology comfort × age, technology comfort × race and/ethnicity, internet access × age, and internet access × race and ethnicity. All the categorical variables were presented using reference coding. ORs with Wald 95% CI values were reported for the key and interaction effects. The fit of the model was determined by −2 log likelihood, Akaike information criterion, and Schwarz criterion, while predictive discrimination was assessed by using the concordance statistic (c-statistic). Missing data were managed by complete-case analysis, where 8 participants were excluded due to missing values.

Sensitivity analyses were conducted to test the stability of the major conclusions. The procedures for the sensitivity analyses included the inclusion of age as a continuous measure, testing alternative categorizations of race and ethnicity, the use of standard LR models without Firth’s correction, and multiple imputation methods to handle covariate data. Additional interaction terms, including technology comfort with age, race and ethnicity, education, and consent language, were also tested. The conclusions were the same under all these different models, hence the confirmation of the identified associations’ stability and robustness.

### Ethical Considerations

This study was approved by the All of Us institutional review board of the NIH (AoU IRB protocol number 2016‐05). All participants provided informed consent online for data linkage and voluntary genomic testing. To protect participant privacy and confidentiality, all data collected were deidentified prior to analysis. Unique identifiers were assigned to securely link survey responses, EHR data, and biospecimen samples while preventing the disclosure of personal information. Participants who completed the primary consent, EHR consent, return of genomic results consent, all 3 enrollment surveys, and physical measurements and provided biospecimen samples received a US $25 gift card as compensation for their time and contribution to the program.

## Results

### Participant Characteristics

There were 605 participants, 64.5% (390/605) of whom were women. Most participants were contained within the group aged 50‐59 years (172/605, 28.4%) and 40‐49 years (111/605, 18.4%). The majority specified their ethnicity as Hispanic (343/605, 56.7%). For race, participants reported White (182/650, 30.1%) and Black (66/605, 10.9%) as the 2 highest categories. Most specified the highest level of education as high school (191/605, 31.6%).

The majority reported access to the internet (539/605, 89.1%), whereas 10.9% (66/605) had no access. Nearly 3 quarters of participants (448/605, 74.1%) reported feeling comfortable with computers, 6.3% (38/605) felt neutral, and 19.7% (119/605) felt uncomfortable (Table 1).

**Table 1.** Study participant demographics. A cross-sectional analysis using participant-reported data to examine technology access and comfort and enrollment support preferences among Federally Qualified Health Centers participants. Missing data were minimal (<1% per variable) and handled via complete-case analysis.

Demographics	Values (N^a^=605)	
Age (years), n (%)		
18‐29	97 (16.03)	
30‐39	80 (13.22)	
40‐49	111 (18.35)	
50‐59	172 (28.43)	
60‐69	111 (18.35)	
70‐79	31 (5.12)	
≥80	3 (0.50)	
Ethnicity^b^, n (%)		
Hispanic	343 (56.69)	
Non-Hispanic	262 (43.31)	
Race^b^, n (%)		
Asian	8 (1.32)	
Black	66 (10.91)	
Middle Eastern	1 (0.17)	
Mixed race	76 (12.56)	
Other	2 (0.33)	
White	182 (30.08)	
Native American	7 (1.16)	
Gender identity, n (%)		
Men	207 (34.21)	
Nonbinary	1 (0.17)	
More than 1 gender identity	4 (0.66)	
Transgender	3 (0.50)	
Women	390 (64.46)	
Language, n (%)		
English	422 (69.75)	
Spanish	183 (30.25)	
Education, n (%)		
Never attended school or only attended kindergarten	1 (0.17)	
Grades 1-4 (primary)	25 (4.13)	
Grades 5-8 (secondary)	40 (6.61)	
Grades 9-11 (some high school)	82 (13.55)	
Grade 12 or GED (high school graduate)	191 (31.57)	
College 1-3 years (some college or technical school)	169 (27.93)	
College 4 years or more (college graduate)	66 (10.91)	
Advanced degree (Master’s, doctorate, etc)	31 (5.12)	

a Participants enrolled in the Community Health Center Inc All of Us Research Program (2017‐2023)

b Self-reported and are presented as underrepresented versus nonunderrepresented for analysis purposes.

### Descriptive Results

Table 2 depicts the correlation among internet accessibility, technological proficiency, and the participants’ inclination toward either a High-Touch or Low-Touch approach to the completion of consent. These factors are examined within the context of the Digital Divide Framework, highlighting both accessibility (internet connectivity) and competencies or comfort (digital literacy) as essential determinants of the enrollment method.

**Table 2.** Distribution of internet access and comfort with technology by consent interaction level (High-Touch, Low-Touch, and NA). Bivariate analysis between access and comfort with technology and enrollment support preference was conducted. Missing data were excluded from analyses (complete-case).

Bivariate analysis^a^	High-Touch^b^ (n=346)	Low-Touch^c^ (n=259)	Test statistic	*P* values
Access to internet, n (%)				
Yes	296 (85.6)	243 (93.8)	10.4	<.005
No	50 (14.5)	16 (6.2)		
Comfort with technology, n (%)				
Comfortable	211 (61)	237 (91.5)	78.1	<.001
Neutral	26 (7.5)	12 (4.6)		
Uncomfortable	109 (31.5)	10 (3.9)		

a Chi-square tests were used to examine bivariate associations between internet access and technology comfort and consent preference.

b High-Touch indicates preference for in-person or staff-assisted consent.

c Low-Touch indicates preference for self-guided consent.

Of those needing High-Touch support (n=346), 14.5% (n=50) had no internet access and 31.5% (n=109) felt uncomfortable with technology. In comparison, from the Low-Touch group (n=259), only 6.2% (n=16) had no internet access and 3.9% (n=10) felt uncomfortable with technology (*P*<.001). These trends are also aligned with the constructs from UTAUT, namely the performance expectancy and effort expectancy on the take-up of technologies, where usability comfort from digital platforms and subjective ability to access them are antecedents to the uptake of Low-Touch channels. The chi-square test also determined that the type of support was significantly correlated with access to the internet (*P*<.005) and feeling uncomfortable with technology (*P*<.001).

### Unadjusted and Adjusted Association Between Internet Access, Technology Comfort, and Completion of Consent Method Preference

Since a few cells existed for some categories, penalized maximum likelihood estimates, including Firth’s correction for bias, were conducted by LRs. The adjusted model contained covariates age, race and ethnicity, education level, and preferred consent language so that the potential confounding variables may be adjusted. Two-way interactions, namely, technology comfort × age, technology comfort × race/ethnicity, internet access × age, and internet access × race and ethnicity, were tested but found not to be statistically significant (all *P*>.24), thus suggesting the absence of moderation effects. All the categorical variables were coded by reference coding. ORs, together with their Wald 95% CI values, were presented for the main effects as well as the interaction effects.

As evidenced by the data included in Table 3, the unadjusted outcome indicated that participants with internet access had significantly lower chances of using the High-Touch method compared to their counterparts without internet access (OR 0.390, 95% CI 0.217-0.702; *P*<.005). Furthermore, participants who were neutral or comfortable with computers had reduced chances favoring the High-Touch method compared to participants uncomfortable with computers (comfortable: OR 0.082; *P*<.001 and neutral: OR 0.199; *P*<.005).

**Table 3.** Logistic regression model: unadjusted and adjusted odds ratios for access to the internet and comfort with technology in relation to use of the High-Touch method for consent completion. Models adjusted for age, race and ethnicity, education, and consent language. Analysis used Firth bias correction for small-cell adjustment.

Variables^a^	Values (N=605)		*P* value
Access to internet, unadjusted OR^b^ (95% CI)			
Yes	0.390 (0.217-0.702)		<.005
No	Reference		
Comfort with technology, unadjusted OR (95% CI)			
Comfortable	0.082 (0.042-0.160)		<.001
Neutral	0.199 (0.077-0.510)		<.005
Uncomfortable	Reference		
Access to internet, adjusted OR (95% CI)^c^			
Yes	1.029 (0.498-2.125)		Not significant
No	Reference		
Comfort with technology, adjusted OR (95% CI)^c^			
Comfortable	0.118 (0.055-0.255)		<.001
Neutral	0.212 (0.077-0.587)		<.005
Uncomfortable	Reference		

a Findings reflect the relationship between structural (internet access) and cognitive (technology comfort) dimensions of the Digital Divide Framework. The adjusted model included age, race and ethnicity, education, and consent language as covariates to account for potential confounding. Penalized logistic regression using Firth bias correction was applied due to sparse cells in some categories. Interaction terms between technology comfort × age, technology comfort × race and ethnicity, internet access × age, and internet access × race and ethnicity were tested but were not statistically significant (all *P*>.24). Missing covariate data were handled via complete-case analysis (8 participants excluded). Sensitivity analyses were conducted and confirmed the robustness of the primary findings.

b OR: odds ratio.

c Covariates included in adjusted models: Age (18‐29, 30‐39, 40‐49, 50‐59, 60‐69, 70‐79, ≥80 y), race and ethnicity (underrepresented vs non–underrepresented), education (1-8), and consent language (English vs Spanish).

After adjustment for covariates (Table 3), the correlation for internet access with preference for High-Touch was no longer significant (AOR 1.029, 95% CI 0.498-2.125; *P*=.94). Comfort with tech was still significant (AOR comfortable 0.118 and AOR neutral 0.212). This outcome highlights the point that digital proficiency and self-confidence (both key elements within the Digital Divide Framework) are superior predictors over access alone, as suggested by TAM and UTAUT, where behavioral intent and adoption are driven by perceived usability ease and confidence with one’s ability to use tech.

Model fit was estimated by Akaike information criterion, Schwarz criterion, and −2 Log Likelihood, respectively. The predictive discrimination was examined by the c-statistic (concordance index). The model diagnostics revealed a good fit. The interaction terms were not significantly different from 0 (all *P*>.24), so the relations did not differ across subgroups.

### Sensitivity Analyses

As an additional test of the robustness of our results, we carried out a number of sensitivity analyses. Results were concordant with the main models, with technology comfort still significantly associated with preference for High-Touch consent and internet access still not significant after adjustment. The replication of technology comfort as a predictor strengthens the theoretical correspondence to TAM and UTAUT constructs, emphasizing the importance of end user’s perceived ability (effort expectancy) for the preference for digital over human-mediated channels. Furthermore, we contrasted complete-case analysis with multiple imputation for missing covariate data and saw equivalent effect estimates by approach. Finally, we estimated standard LR without Firth’s correction and tested interaction terms one-by-one. These alternative specifications did not substantially alter the identified associations. Altogether, the combined set of analyses suggests that our results are stable and robust to different analytic assumptions as well as variable codings.

## Discussion

### Summary of Main Findings

In alignment with the study objectives, this study examined support preference with digital access and comfort with technology levels for large-scale enrollment in research. Although most participants had internet access (539/605, 89.1%), technological comfort, represented by ease of use in TAM and effort expectancy in UTAUT, was the primary driver of High-Touch compared to Low-Touch preference. Those reporting discomfort with technology were significantly more likely to choose a High-Touch (staff-mediated) enrollment support preference. Although unadjusted analyses implied lack of internet access was related to High-Touch support preference, this relationship was no longer significant when adjusted for covariates age and race/ethnicity. These results extend the Digital Divide framework from connectivity, including user skill, confidence, and behavioral intention as key determinants of behavior [26-28].

### Interpretation and Comparison to Existing Literature

Findings from this study suggest substantial implications for digital health research infrastructure. Continued access to device and broadband service is needed, but the TAM and the UTAUT models indicate enrollment behavior is stronger when the participant’s perceived ease of use (comfort level) and the influence of others (endorsements from staff members or colleagues) are present compared with access only. This is consistent with emerging evidence demonstrating digital self-efficacy, rather than access alone, is the greatest predictor of participation in the digital healthcare and research space [29,30].

The decreased significance for internet access in the adjusted models may also be an indication of the effect of the sociodemographic covariates. For instance, participants without internet access were frequently older or from underrepresented populations, which were already accounted for within the adjusted models. Again, the relatively small group of participants without internet access may have also limited statistical power. This highlights the point that the noted absence of significance does not necessarily mean no effect but instead suggests an achievable contextual or sample-specific restriction. This supports the need to consider both access characteristics as well as user characteristics within strategies to foster digital inclusion.

The findings from this study are consistent with other studies that show overlap of digital literacy with ease of convenience, trust, and privacy, especially among underrepresented groups in biomedical studies. By implicitly correlating the above results with TAM and UTAUT and Digital Divide frameworks, this study clarifies the mechanisms through which digital comfort impacts research enrollment. Hannemann et al [27] discussed participants’ typology from “digital skeptics” to “digital illiterates,” with the standard of nonparticipation generally founded upon ease of convenience rather than accessibility. Similarly, Batterham et al [28] showed that significant proportions of participants placed importance upon trust and support ahead of convenience, especially for taking on difficult health-related processes through the internet. With projects like the Biobank of the UK or the AoURP, there has also been increased application of self-directed digital tools of consent with improved scalability; however, there is a risk of excluding nondigitally literate consumers [31-33]. Our study adds to this notion by suggesting that mere access to the internet does not fully explain support preference. Instead, functional comfort with technology is a stronger predictor, highlighting a potential gap in prior work that emphasizes connectivity alone. Hybrid consent models, where remote tools are supplemented with human facilitation, appear aligned with digital equity models that advocate for context-aware and culturally sensitive systems [31,32,34,35].

Digital enrollment processes for precision medicine studies like AoURP should include TAM and UTAUT principles of perceived ease of use, an index of technological comfort, together with performance expectancy, the belief in one’s ability to perform things by oneself. One example is the use of short digital literacy tests within consent procedures, allowing for the allocation of potential participants into either the Low-Touch or the High-Touch track. Subjects with low digital comfort level are offered automated human assistance or alternative means of consent, like videoconferencing or face-to-face assistance [11,33,36,37]. These participant-oriented design principles support the main principles of digital equity in terms of meeting participants where they are, both experientially and technologically [35]. They are also backed by research findings indicating that there is the possibility of technical discomfort causing withdrawal, mistrust, or outright refusal of research access, even when barriers to access have been overcome [13,14,36,37].

### Implications for Research Design and Practice

The significant correlation between the level of technological comfort and the High-Touch enrollment preference is an indication that factors such as confidence among staff, past experiences, and cultural orientations have the potential to serve as mediators on effort expectancy and intentions to behavior, as the UTAUT model suggests. Specifically, factors such as confidence among research staff members, research staff’s past experience with digital technologies, or cultural orientations toward digital technologies are likely to have potential as mediators on the relationship between the level of technological comfort and enrollment preference for High-Touch support. High-Touch designs, characterized by guided support via telephone, videoconferencing, or face-to-face interactions, are essential for facilitating engagement from participants who are hesitant to use technology. To translate these insights into practice, FQHCs and other health centers could develop flexible enrollment pathways that include (1) a concise digital literacy assessment during the intake process, (2) automatic direction to either Low-Touch or High-Touch support based on the assessment outcomes, (3) training and use of digital navigators to offer immediate assistance, and (4) monitoring of enrollment results to continuously refine the triage process. Success for implementing this framework in practical settings depends on the development of triaged workflows, evaluations of digital literacy, the use of digital navigators, and a combination of support options, all of which collectively seek to bridge the digital divide.

### Strengths and Limitations

The study capitalizes on real-world data regarding internet access and technology comfort from patients covered by an FQHC. However, this study does have its limitations. First, the analysis only contained participants with complete question answers from the survey, raising the possibility of selection bias if the participants with missing data had different levels of internet access or technology comfort. Second, the study was conducted by means of a single-site study, so generalizability to other sites, for example, rural clinics or academic medical centers, is limited. This study dichotomized support preference as High-Touch versus Low-Touch paths, but enrollment engagement is well-known to exist on a continuum and not as dichotomies, with some participants using a hybrid of different strategies. It was not possible with the current dataset to empirically test the evaluation of the hybrids of enrollment engagement models, an important limitation. Quantification of gradient support strategies with subsequent measurement of their performance among participant subgroups will become an important area of future studies, allowing for better nuance and adaptability of digital enrollment systems.

Another study weakness is the absence of an a priori power calculation, as the survey was based on existing program enrollment data rather than the prospectively determined sample. Although the overall sample was sizable, subgroup samples, such as those without internet access, were small. Thus, the nonsignificant outcome for the adjusted relationship between internet access and High-Touch preference may indicate an index of poor statistical power rather than an index of the true null effect. Furthermore, the statistically nonsignificant adjusted relationship between internet accessibility and High-Touch preference could indicate poor statistical power or adjustment for covariates instead of a genuine null effect. Potential confounding factors such as previous digital training, proficiency with language, health literacy, or cultural orientations toward technology are able to influence both comfort and support preference. Their omission may impede causal inference.

Finally, the reliance on self-reported measures for technology comfort presents the potential for biases toward social desirability and recall, whereas the cross-sectional design precludes the possibility of making causal inferences. Despite these limitations, the study contributes valuable insight into the relative importance of comfort with technology relative to limited access to the internet, informing the dichotomy between High-Touch versus Low-Touch approaches to researching enrollment. In addition, despite the limitations, the conceptual synthesis provides improved insight into the influence associated with digital literacy with user-friendliness on enrollment preference over access.

### Conclusions and Broader Implications

This study demonstrates that participants’ level of tech-savviness, more than internet access, has a significant influence on their preference toward High-Touch instead of Low-Touch enrollment practices in large-scale digital health research. These outcomes contribute toward existing work on the digital divide by underscoring that participation barriers are determined by an intersection between digital expertise and psychosocial constructs, including trust and previous experiences with online technologies.

From a research perspective, these results point out the need to consider the measure of technological comfort, digital expertise, and psychosocial mediators during the design of enrollment procedures. From a policy perspective, funding and guidance for digital health projects should prioritize flexible, participant-centric enrollment models that are able to accommodate varied levels of technological comfort and digital expertise.

In practice, FQHCs and other similar settings can implement the following achievable strategies: (1) triaged enrollment procedures, (2) concise digital screenings during the intake process, (3) use of digital navigators, and (4) hybrid support methods combining digital portal access with human assistance. Programs must also track outcomes from an enrollment perspective by level of comfort among the participants and provide multiple opportunities for the development of skills so that the process is equitable.

Based on these findings, future research is necessary to build and test frameworks permitting participant interaction with research initiatives like the AoURP. Additional research is also necessary to test potential mediators, including research staff trust, histories of online exposure, and cultural orientations to technologies, to assess their effect on the interdependence between technological comfort and support preferences. Sensitivity analyses, as well as alternative specifications for the model, must be done to test the robustness of the conclusions, particularly among the subgroup that has limited access.

Hybrid enrollment procedures combining digital portal and human-assisted methods need testing, especially for FQHCs or low-resource sites. Some concrete strategies may include the addition of short digital literacy screenings during intake procedures, the adoption of trained staff as digital navigators, and the provision of on-demand support by one-on-one, phone, or video. Hybrid or mixed enrollment strategies also need to be tested empirically so the flow of participants between the Low-Touch and the High-Touch paths is better understood, as well as whether different combinations offer improved retention and data quality.

Additional research is also needed to assess interventions for enhancing digital confidence and digital literacy among the research participants, their families, and the underrepresented group members. Modular digital skills training, digital mentoring by the community, as well as staff-piloted mobile technology toolkits by the FQHC staff, are some of the interventions to be included. Completion rates of enrollment, retention of the participants, data quality, as well as the level of comfort the technology provides to the participants should all be included as measurable outcomes.

Subsequent studies must incorporate digital literacy as a routine survey question so that inclusion trends are measurable over time. Mixed-method studies should test mediators (previous experience, cultural attitudes, and trust) and test adaptive, participant-centered enrollment strategies. Long-term end points of the interventions will inform a scalable, equity-focused digital health research infrastructure.

Future research should also address these specific, measurable questions:

1. Does digital literacy initiative participation contribute to the increase in Low-Touch enrollment pathway selections?
2. Does trust in staff, prior online experience, or cultural perceptions mediate the association between technology comfort and support preference?
3. How do sociodemographic variables influence the effectiveness of mixed enrollment strategies and by what mechanisms?
4. What is the impact of digital navigators combined with adaptive support on enrollment completion, retention, and data quality?

The combined implementation of these approaches can make digital health research infrastructure scalable, fairer, and more inclusive. This assists in getting all stakeholders meaningfully involved, particularly members from disadvantaged groups, in precision medicine activities.

## Supplementary material

10.2196/78850 Checklist 1 STROBE checklist.

## Abbreviations

AoURP: All of Us Research Program
EHR: electronic health record
FQHC: Federally Qualified Health Center
LR: logistic regression
NIH: National Institutes of Health
OR: odds ratio
STROBE: Strengthening the Reporting of Observational Studies in Epidemiology
TAM: technology acceptance model
UTAUT: unified theory of acceptance and use of technology
