# Supplementary material for: Technology Access, Digital Literacy, and Enrollment Support Preferences in a Federally Qualified Health Center: Cross-Sectional Study
Source: JMIR Form Res. 2026 Jan 5;10:e78850. doi: 10.2196/78850 (PMC12767775; doi:10.2196/78850)
Supplement: Checklist 1 [file formative-v10-e78850-s001.pdf]

STROBE Statement—Checklist of items that should be included in reports of ***cross-sectional studies***

STROBE Statement—Checklist of Items That Should Be Included in Reports of Cross-Sectional Studies

**Section / Topic**	**Item No**	**Recommendation**	**Location in Manuscript (page/paragraph or section)**
**Title and Abstract**	1a	Indicate the study's design with a commonly used term in the title or the abstract	Title: "A Cross-Sectional Study"; Abstract, first sentence under *Methods*
	1b	Provide in the abstract an informative and balanced summary of what was done and what was found	Abstract, all subsections (Background, Objective, Methods, Results, Conclusions)
**Introduction**	2	Explain the scientific background and rationale for the investigation being reported	Introduction, paragraphs 1–3
	3	State specific objectives, including any prespecified hypotheses	Introduction, final paragraph (explicit hypothesis added)
**Methods**	4	Present key elements of study design early in the paper	Methods, first subsection ("Study Design")
	5	Describe the setting, locations, and relevant dates, including periods of recruitment, exposure, and data collection	Methods, *Study Setting* subsection (CHCI, Connecticut, 2017–2023)
	6a	Give the eligibility criteria and the sources and methods of selection of participants	Methods, *Participants* subsection
	7	Clearly define all outcomes, exposures, predictors, potential confounders, and effect modifiers	Methods, *Variables* subsection
	8	For each variable of interest, give sources of data and details of methods of assessment (measurement)	Methods, *Measures* subsection
	9	Describe any efforts to address potential sources of bias	Methods, *Measures* and *Strengths and Limitations* sections
	10	Explain how the study size was arrived at	Methods, *Sample Size and Power Considerations*
	11	Explain how quantitative variables were handled in the analyses	Methods, *Analysis* subsection
	12a	Describe all statistical methods, including those used to control for confounding	Methods, *Analysis* subsection
	12b	Describe any methods used to examine subgroups and interactions	Methods, *Analysis* subsection (interaction terms specified)
	12c	Explain how missing data were addressed	Methods, *Analysis* subsection (complete-case analysis)
	12d	If applicable, describe analytical methods taking account of sampling strategy	Not applicable (single-site census sample)
	12e	Describe any sensitivity analyses	Methods, *Sensitivity Analyses* subsection
**Results**	13a	Report numbers of individuals at each stage of study	Results, *Participant Characteristics* section

Section / Topic	Item No	Recommendation	Location in Manuscript (page/paragraph or section)
	13b	Give reasons for non-participation at each stage	Results, *Participants* section (noted exclusions for missing data)
	13c	Consider use of a flow diagram	Not applicable—simple single-stage recruitment described in text
	14a	Give characteristics of study participants and information on exposures/confounders	Results, *Table 1* and narrative text
	14b	Indicate number of participants with missing data for each variable	Results, footnotes to Table 1
	15	Report numbers of outcome events or summary measures	Results, Tables 2–3
	16a	Give unadjusted and adjusted estimates and precision (95% CI), specifying confounders	Results, *Table 3* and narrative text
	16b	Report category boundaries when continuous variables were categorized	Methods, *Variables* subsection
	16c	If relevant, translate relative risks into absolute risks	Not applicable (no time-to-event or risk measures)
	17	Report other analyses (subgroups, sensitivity analyses)	Results, *Sensitivity Analyses* subsection
**Discussion**	18	Summarize key results with reference to study objectives	Discussion, *Summary of Main Findings* subsection
	19	Discuss limitations of the study, considering sources of bias or imprecision	Discussion, *Strengths and Limitations* subsection
	20	Provide cautious overall interpretation of results considering objectives and prior evidence	Discussion, *Interpretation and Comparison to Existing Literature* subsection
	21	Discuss generalisability (external validity) of the study results	Discussion, final paragraph before Conclusions
**Other Information**	22	Give the source of funding and the role of the funders for the present study	*Funding Statement*, page 22 of manuscript
